# Microwave-assisted catalysis of water-glycerol solutions for hydrogen production over NiO/zeolite catalyst

**DOI:** 10.1016/j.heliyon.2021.e07557

**Published:** 2021-07-12

**Authors:** Husni Husin, Mahidin Mahidin, Komala Pontas, Ahmadi Ahmadi, Muhammad Ridho, Erdiwansyah Erdiwansyah, Fahrizal Nasution, Fikri Hasfita, M. Hazwan Hussin

**Affiliations:** a Reaction Engineering and Catalysis Laboratory, Department of Chemical Engineering, Faculty of Engineering, Universitas Syiah Kuala, Jl. Tgk. Syech Abdurrauf No.7, Darussalam, Banda Aceh, 23111, Indonesia; b Doctoral Program, School of Engineering, Universitas Syiah Kuala, Darussalam, Banda Aceh, 23111, Indonesia; c Department of Chemical Engineering, Faculty of Engineering, Universitas Malikussaleh, Lhokseumawe, Indonesia; d Faculty of Engineering, Universitas Serambi Mekkah, Banda Aceh, 23245, Indonesia; e School of Chemical Sciences, Universiti Sains Malaysia, 11800, Minden, Penang, Malaysia

**Keywords:** Microwave, Glycerol, Hydrogen energy, NiO/zeolite

## Abstract

In this study, glycerol as an abundant green feedstock was used as a hydrogen source to investigate the reaction of water-glycerol solution decomposition by microwave-assisted catalytic to produce hydrogen over NiO/zeolite catalyst. The catalyst was prepared by inception wetness and then characterized through X-ray diffraction (XRD), scanning electron microscopy (SEM), Energy diffraction X-ray (EDX), and transmission electron microscope (TEM) measurements. The conversion process of glycerol into hydrogen was performed in a fixed-bed microwave-assisted reactor. Effect of microwave power, NiO content, and feed flow rate (FFR) on glycerol conversion and hydrogen selectivity were studied. The results of XRD and EDX measurement showed that NiO crystalline exists on the catalyst sample. The particle size of NiO/zeolite was determined in the range of 30–300 nm, and the particle was found well dispersed on the zeolite surface as confirmed by TEM. Furthermore, the maximum conversion rate can achieve about 96.67 %, while the highest hydrogen production was found up to 73.5 % with the condition of 20% of NiO as an active site on natural zeolite. It was found that the NiO content of 20% gave the best glycerol conversion at the microwave power of 600 W and FFR 0.5 ml/min. Microwave-assisted catalytic irradiation of glycerol appears to be a promising candidate for the production of H_2_ from an aqueous glycerol solution.

## 1 Introduction

Sustainable energy and a clean environment are two important concerns of modern society nowadays, although, in fact, the primary source of energy still depends on fossil fuels [1, 2, 3]. Declining oil reserves and increasing environmental issues have stimulated energy experts to seek a new alternative fuel sources to replace petroleum-based fuels [4, 5, 6]. A pressing need has thus emerged to exploit alternative energy sources, i.e., energy that will be both renewable and environmentally friendly [7, 8].

Hydrogen is one of the energies which can be produced from clean and renewable sources [9]. Renewable hydrogen sources are known as secondary energy since they can replace fossil fuels in generating heat or electricity for the combustion systems [10]. This idea has attracted the attention of scientists to discover a new feedstock that is renewable and sustainable [11, 12]. Currently, it faces two serious problems: reduced raw materials and environmental pollution resulting from its use. Based on its importance, researchers have attempted to investigate many methods to produce hydrogen and searching the raw materials [13, 14, 15, 16, 17].

Several studies have studied the use of renewable sources such as water and glycerol [18], methanol [19], ethanol [20] (biomass derivatives) to produce hydrogen with alternative technologies that are cheaper, energy-efficient, and environmentally friendly. Considering the large source of glycerol, a byproduct from the biodiesel industry, it will be worthwhile to utilize renewable sources for hydrogen production [21, 22]. To this end, waste biodiesel, with its high source of 10 wt.% glycerol [25], is a promising raw material for the production of hydrogen. Producing H_2_ from glycerol is another approach that is being investigated in this work. Several processes have been reported to convert glycerol into hydrogen, such as steam reforming process [23, 24, 25], pyrolysis [26], photocatalytic [27], and microwave heating [28]. The glycerol steam reforming process takes place according to the following stoichiometric equation:

(1) C_3_H_8_O_3_ + 3H_2_O → 3CO_2_ + 7H_2_ (ΔH^o^) +346.4 kJ/mol)

Currently, most of the works have been carried out via steam reforming and water electrolysis. The drawback of this reaction requires large amounts of energy [29]. Still, it uses fossil-based materials as fuel in its process. In addition, the process also produces pollutants as a source of greenhouse gas emission, i.e., CO_2_ from steam reforming reaction can contribute to the global warming effect. Besides, the water electrolysis method uses renewable sources [30], which require a large amount of electrical energy. The high price of raw material cause to lead the overall processing cost and makes the process uneconomical [31].

Microwave irradiation is one of the most promising methods to accelerate the reaction in the catalytic area [32]. The microwave-assisted catalytic (MAC) offers many advantages with the conventional heating process, including internal fast and uniform heating of all solutions, no need for solution agitation, rapid start-up and ease of control, and energy cost saving. Microwave irradiation can decrease reaction time from hours to minutes. During the heating process, the rapid rise of temperature by rapid rotation of dipole by the electric field and catalyst is the key to the fast increase in chemical reaction rates by using microwave [33, 34, 35]. Shi et al. [36] employ a microwave-assisted combination with carbon as a catalyst to accelerate reaction biomass into hydrogen energy. The yield of gaseous products was obtained, ranging from 73 to 76 wt.% at 600–800 °C, and a hydrogen product reaches 55.7 vol.%. Deng et al. [37] have successfully applied the MAC technique to process ethanol decomposition over residue of sewage sludge catalyst to produce hydrogen. The microwave was adjusted from 300-560 W. The reaction in the microwave is much more beneficial than conventional heating. The ethanol conversion increased with the increase of microwave power. The ethanol conversion reaches 98.4% at optimum temperature. In a previous study, Fernandez et al. [38] investigate pyrolysis of pure glycerol using carbonaceous catalysts in a microwave. The experiments were performed at 400, 500, 600, 700, 800, and 900 °C, produce the synthesis gas up to 81 vol.%.

Usually, to accelerate the reaction rate of hydrogen production, noble metal-based catalysts are frequently used and present high activity. However, it offers some disadvantages, e.g., it promotes the coke formation at high temperature [39]. Nickel-based [40, 41, 42] are the most promising catalyst, high activity, and low-cost compared to noble metals [43, 44]. Our previous work [45] employed nickel on NaTaO_3_ for photocatalytic methanol and glycerol/water systems to hydrogen production. Based on literature study, a microwave-assisted water-glycerol system combined with a NiO/Zeolite catalyst for hydrogen production has been further developed. NiO-supported zeolite (NiO/Zeolite) catalysts were used as an active site in this work. Nickel is acting as an active site which leads to significantly accelerates hydrogen production from water in the presence of glycerol as a sacrificial electron donor. Although microwave technology has been investigated for biofuel production, so far, limited literature studies were found to report on the effect of the NiO/Zeolite catalyst over microwave heating to produce hydrogen from water-glycerol solutions are very limited explored.

This study aims to investigate a detailed examination of microwave heating on the decomposition of water-glycerol solution into hydrogen over NiO/zeolite catalyst. The microwave setup combines with NiO/Zeolite catalysts to improve reforming reaction in terms of conversion and selectivity. For this purpose, nano-catalyst NiO/Zeolite was synthesis via the inception wetness method. NiO/Zeolite catalysts were characterized by various techniques such as XRD, SEM, TEM, and EDX. The effects of operating parameters such as microwave power, feed flowrate, and also the loading NiO/Zeolite were investigated in detail.

## 2 Experiments

### 2.1 Materials

The natural zeolite used in this study was collected from Ujung Pancu, Banda Aceh, Indonesia. Prior to use, the crushed natural zeolite was placed into the oven to reduce the water content. The Ni(NO_3_).6H_2_O (Merck), AgNO_3_ (Merck), Glycerol, and HCl (Merck) were purchased from PT. Rudang Jaya, Medan, and used as received. Nitrogen (N_2_) and oxygen gases with purity 99.0% were purchased from a local Gas Company, Serikat Gas, Banda Aceh, Indonesia. Distilled water was supplied from Chemical Engineering Development Laboratory at Syiah Kuala University, Banda Aceh, Indonesia.

### 2.2 Preparation of natural zeolite

Natural zeolites were crushed and sieved with a 250-mesh sieve of particle size. The particle was washed using distilled water and dried at a temperature of 120 °C. Zeolite was activated by inserting it into 3M of HCl solution. The mixture was filtered and washed with distilled water until was neutral and showing a negative test to the presence of Cl-ions using an AgNO_3_ solution. The residue was dried in an oven at temperatures of 100–120 °C. The dried zeolite was sieved again until a homogeneous zeolite was obtained. The sample was labelled as acid-activated natural zeolite.

### 2.3 NiO/zeolite catalyst preparation

Nickel as an active site was loaded on the surface zeolite powder to accelerate conversion of reactant and promote H_2_ production. To evaluate the effect of catalyst NiO: Zeolite content on conversion and selectivity, in brief, the Ni(NO_3_)_2_.6H_2_O (Merck; 99.0%), eg. 5%, 10%, 15%, and 20 wt% were first mixed in appropriate amount of distilled water. The solution was loaded on zeolite surface through the inception wetness method. The mixture was stirred at a room temperature for six hours. Then the catalyst was dried at 110 °C for 12 h in an oven. The nickel-loaded zeolite was calcined at 500 °C for two hours in the air to form NiO oxide. The dried catalyst was then calcined at a temperature of 500 °C for three hours in a tube furnace. The powder was cooled in the desiccator and store in the glass bottle.

### 2.4 Catalyst characterization

X-ray diffraction (XRD) were measured using a Siemens D5005 diffractometer using Cu-Ka radiation (k = 1.5418 Å and operating at 30 kV and 20 mA). To investigate the morphology of the structure, a scanning electron microscopy (SEM, JEOL JSM-5600L). Elemental composition of the NiO/zeolite was obtained from energy dispersive x-ray spectroscopy (EDS) which was assembled on the SEM instrument. The microstructures were characterized using transmission electron microscope (TEM, JEOL 2010 TEM instrument) USM Malaysia.

### 2.5 Experimental setup and procedure

**Figure 1:**
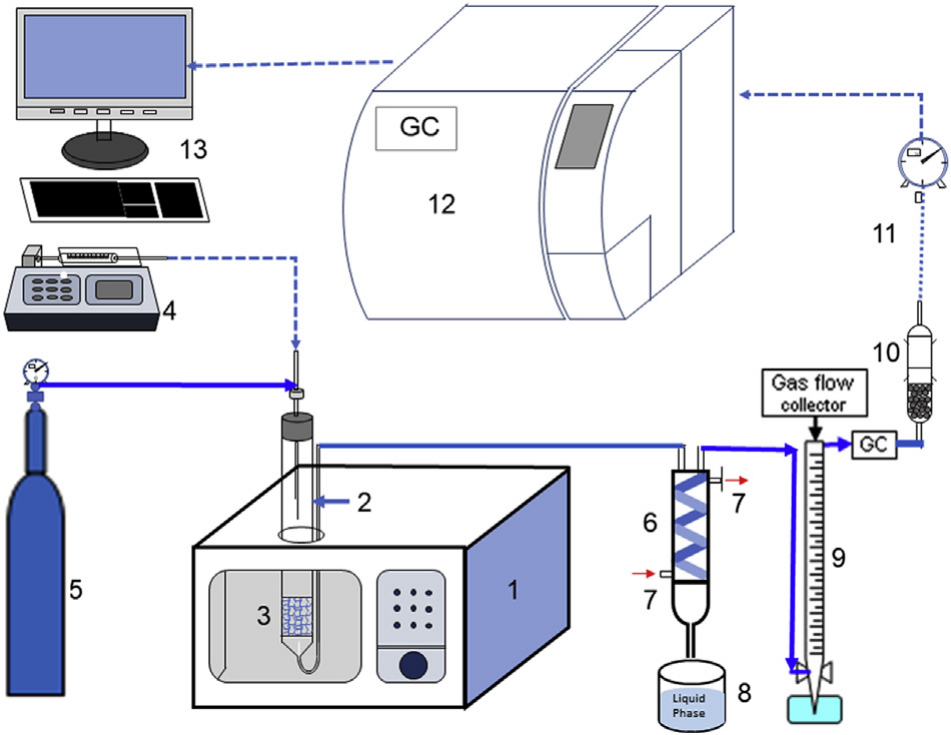
A schematic diagram of microwave-assisted catalytic (MAC) experimental setup (1: microwave oven; 2: quartz glass reactor; 3: catalyst; 4: pump; 5: N_2_ gas; 6: condenser; 7: cooling water; 8: liquid product; 9: gas collector; 10: silica gel; 11: gas flow meter; 12: gas chromatography; 13: recorder).

The reaction of glycerol decomposition for hydrogen generation was carried out in a quartz glass reactor under microwave irradiation. The output power can be manually regulated at 300, 400, 500, and 600W. Feed flow rate was set at 0.25, 0.5, 0.75, and 1.0 ml/min to explore the effect of feed flow rate, microwave heating on conversion and selectivity. All the experimental tests were repeatability of is three times. A quartz glass reactor (40 cm length, 1.0 cm i.d.) filled with 1 g of catalyst was inserted into the microwave oven. Liquid glycerol: water was injected into the reactor with ratio of 1:10. The flowrate of glycerol was precisely controlled by a micro-syringe. Before the microwave started, the air inside the reactor was evacuated by flowing nitrogen as a carrier gas. After a certain time, liquid products were condensed after passing through the condenser. The non-condensable gas product passed through the top of the condenser and headed straight to Gas Chromatography to test gas component was formed. The amount of hydrogen production, which is the test parameter in this study, was measured using gas chromatography (Porapax N column and molecular sieve 5A, PDHID detector with helium gas). The schematic of the conducted reaction system is shown in Figure 1.

## 3 Results and discussion

### 3.1 Catalyst characterization

#### 3.1.1 Diffractometer X-ray (XRD) analysis

**Figure 2:**
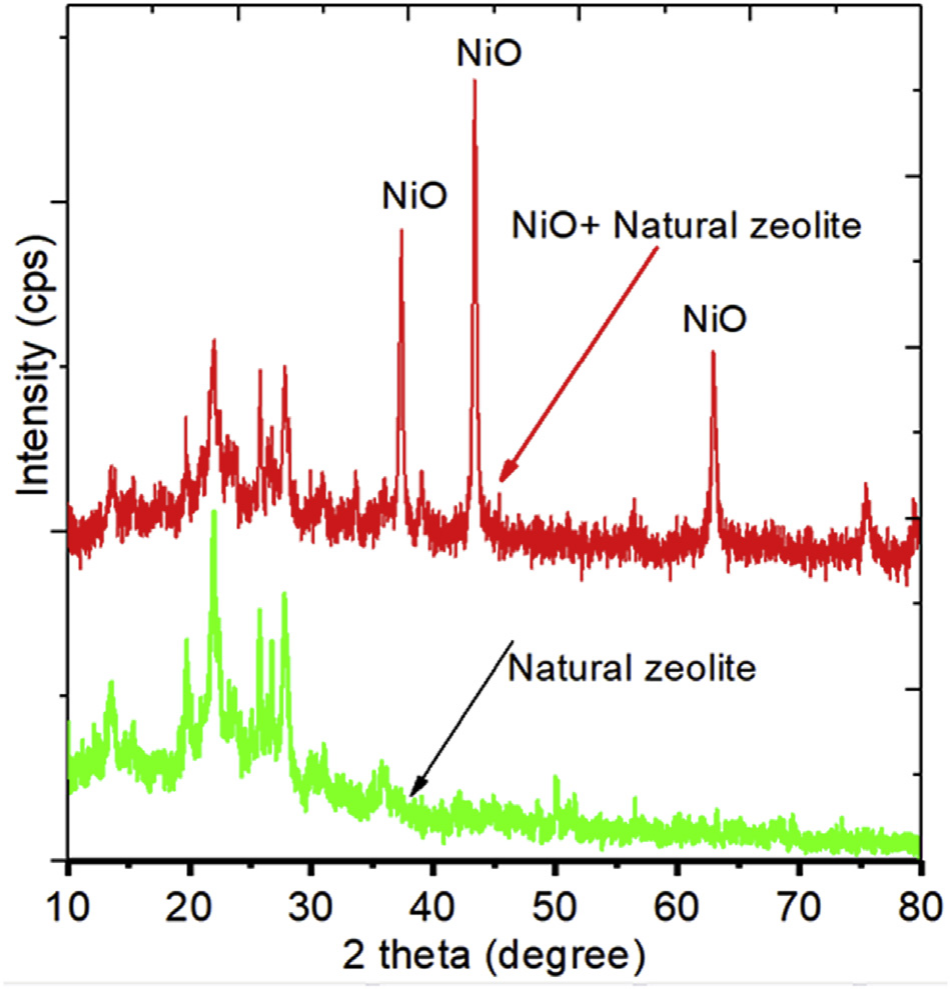
XRD patterns of natural zeolite and NiO/zeolite.

Figure 2 shows an XRD pattern of the NiO/zeolite catalysts. The patterns indicated the formation of a crystalline phase indexed by a single cubic phase (NiO) and a-natural zeolite phase; no crystalline impurities or metallic nickel was observed.

From Figure 2, it can be seen that there was an addition of a few peaks after the impregnation of NiO on the natural zeolite. Peak generated from the catalyst NiO/zeolite was found similar to the primary structure of zeolite diffraction pattern along with some additions of NiO peak. Compared with the calcined zeolite catalyst, there was no significant change in the structure of the natural zeolite, indicating that zeolite has higher thermal stability. The peaks at 37.21^o^, 43.24^o^, and 62.81^o^ indicate the formation of a nickel phase characteristic, which are related to the NiO crystal faces of (111) and (200), respectively. The existence of the NiO on the surface of natural zeolite could attribute to the higher activity of the catalyst for hydrogen production [45].

#### 3.1.2 Scanning electron microscope (SEM-EDX) analysis

**Figure 3:**
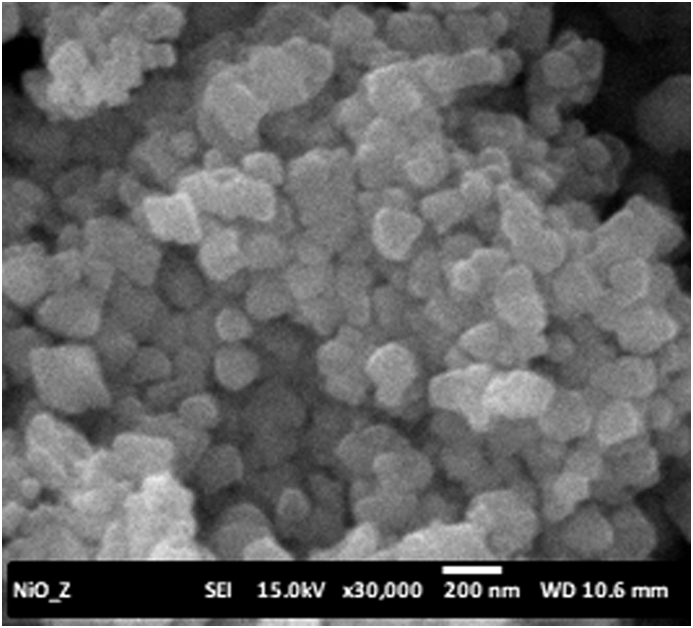
SEM micrograph of NiO/Zeolite catalyst.

SEM-EDX analysis is used to identify the particle size, the structure of the catalyst morphology and the composition of the catalyst atoms, as can be seen in Figure 3.

Figure 3 shows the morphology of the catalyst NiO/Zeolite that has an irregular shape, and some of them approached the square. The white particles of NiO were distributed on the surface of the support and form a reinforced composition. However, the NiO particles did not clearly appear on the support surface. This finding could be the particle size of the zeolite support was in nano size. The catalyst has an average particle size ranging from 50-200 nm. It is worth pointing out that white particle's distribution indicates the metal has succeeded bearer of zeolite surface.

**Figure 4:**
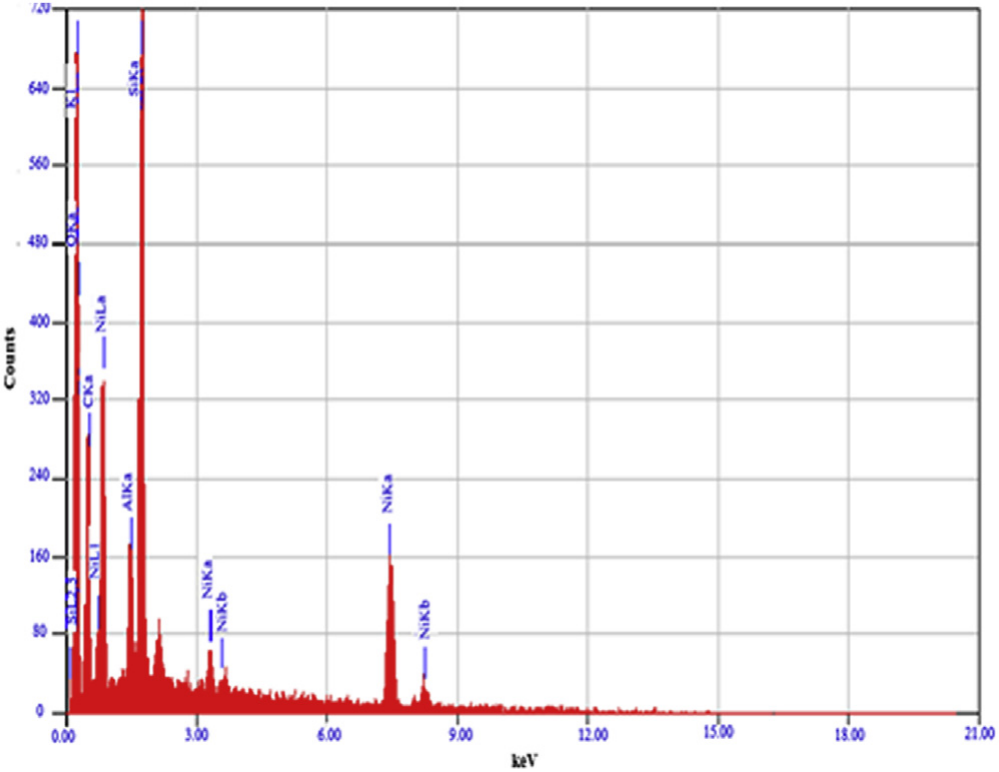
EDX analysis of the elements in the NiO/zeolite catalyst.

The EDX spectra of synthesized NiO/Zeolite nanoparticle are shown in Figure 4 and Table 1. The element content of NiO/zeolite catalyst is shown in Table 1 and Figure 4. It is clearly seen that the NiO/zeolite catalyst is composed from Al_2_O_3_, O, SiO_2_, K_2_O, and NiO, respectively. The elemental analysis by EDX shows that impregnation of NiO on the zeolite support successfully obtained NiO about 56.61%. This fact is in accordance with the SEM, TEM, and XRD data that NiO is successfully produced and deposited on the surface zeolite support. A good distribution of the catalyst is a key requirement to achieve high conversion efficiency for high catalytic sites.

**Table 1:** The content of the elements in the catalyst NiO/Zeolite.

Element	% mass	% atom	Elements/compounds	cations K
O	59.2	73.64	O	16.97
Al	1.94	0.98	Al_2_O_3_	2.84
Si	12.19	11.79	SiO_2_	21.10
K	1.11	0.38	K_2_O	2.48
Ni	28.55	13.21	NiO	56.61

#### 3.1.3 Transmission electron micrographs (TEM) analysis

**Figure 5:**
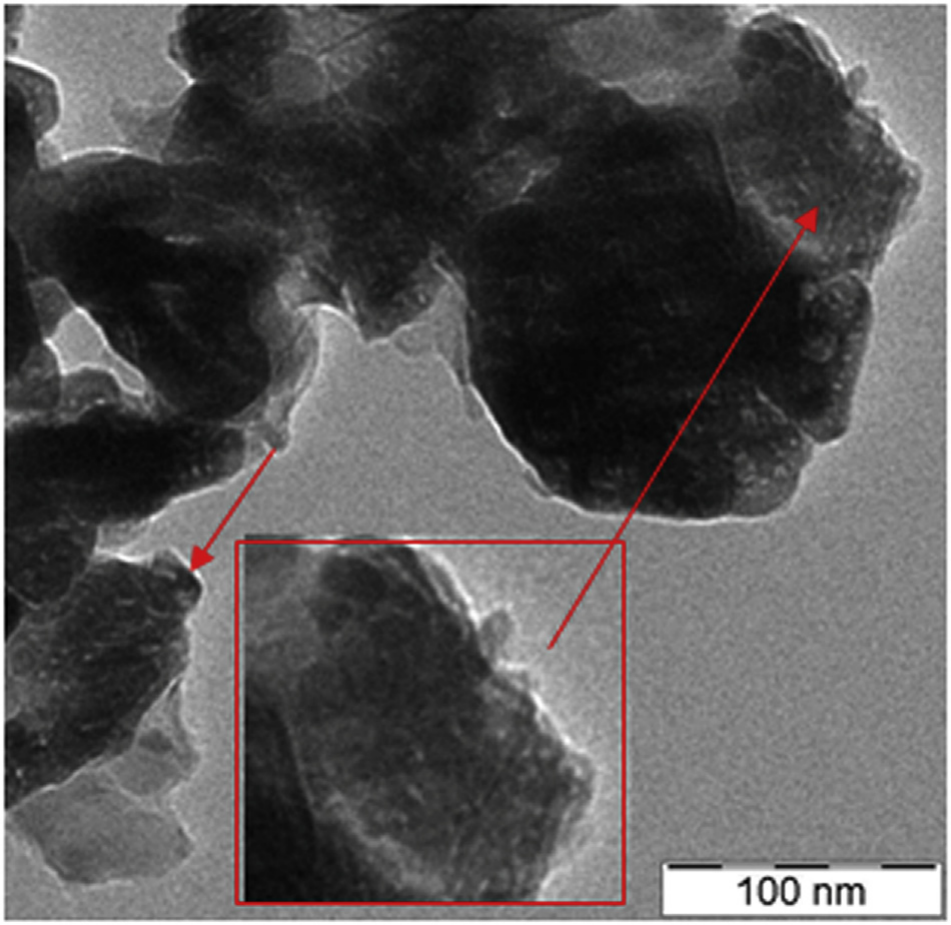
TEM image of NiO/Zeolite catalyst.

Transmission electron micrographs of the NiO/zeolite catalysts are shown in Figure 5. The morphologies of all the catalysts are irregular with slight agglomeration. The particle size of the catalysts is in the range of 30–200 nm, in agreement with the particle size calculated from the SEM image.

The insert picture clearly shows a good dispersion of NiO species on the entire surface of zeolite support. It also indicates that the particle size of NiO around 5 nm has a high dispersion due to the large surface area. These results are consistent with the XRD patterns presented above as well. Based on previous research [46], good catalytic activity is enabled by the preservation of the support material after catalyst preparation. In addition, the metal oxide species must be highly distributed upon the surface of the support.

### 3.2 Catalytic tests of glycerol decomposition

#### 3.2.1 Influence of MAC power on glycerol conversion at different feed flow rate (FFR)

**Figure 6:**
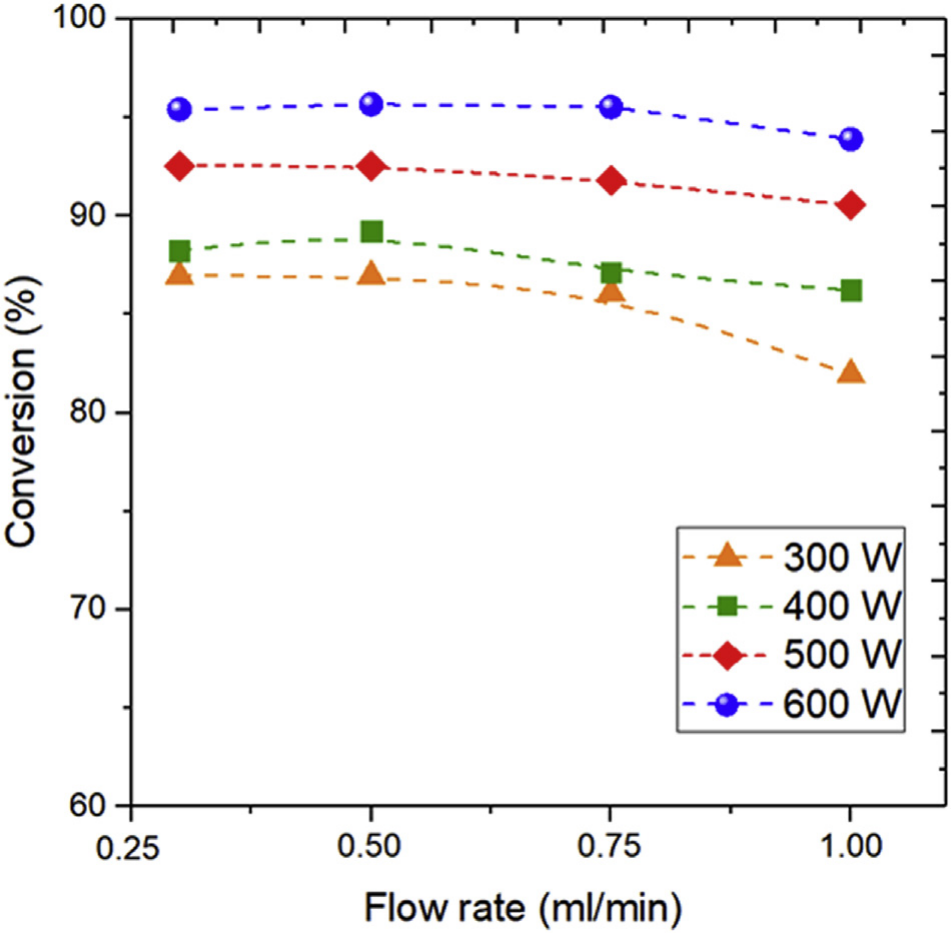
The influence of microwave power and feed flow rate on the glycerol conversion using the catalyst NiO/zeolite of NiO content of 20%.

Figure 6 shows the relationship between the MAC power and flow rate on glycerol conversion. As observed in Figure 6, the glycerol conversion did not reduce with an increase in FFR from 0.3 to 0.5 ml/min. A further increase in FFR from 0.75 to 1.0 ml/min could decrease the glycerol conversion. This finding is in accordance with Deng et al. [39] that the decrease in glycerol conversion was mainly due to reduced resident time.

The microwave power has also a significant influence on glycerol decomposition (Figure 6). An increase in microwave power could increase the glycerol conversion. At a microwave power of 300 W, with a FFR of 0.5 ml/min, glycerol degradation reached 86.95%. Further increase of microwave up to 400 W result increased in glycerol conversion of 89.23%. The higher microwave power gave a greater rate of glycerol conversion. The maximum glycerol conversion was 96.67% for NiO/zeolite loaded 20% at the microwave power of 600 W. According to Hossain et al., the greater power will increase energy supply to the process of water-glycerol degradation. The greater energy that can add to hot spots in the reaction can increase the number of collisions between molecules; hence the catalytic activity and the reaction rate increased the degradation of water-glycerol [32].

#### 3.2.2 Influence of MAC power on glycerol conversion at different NiO loading

**Figure 7:**
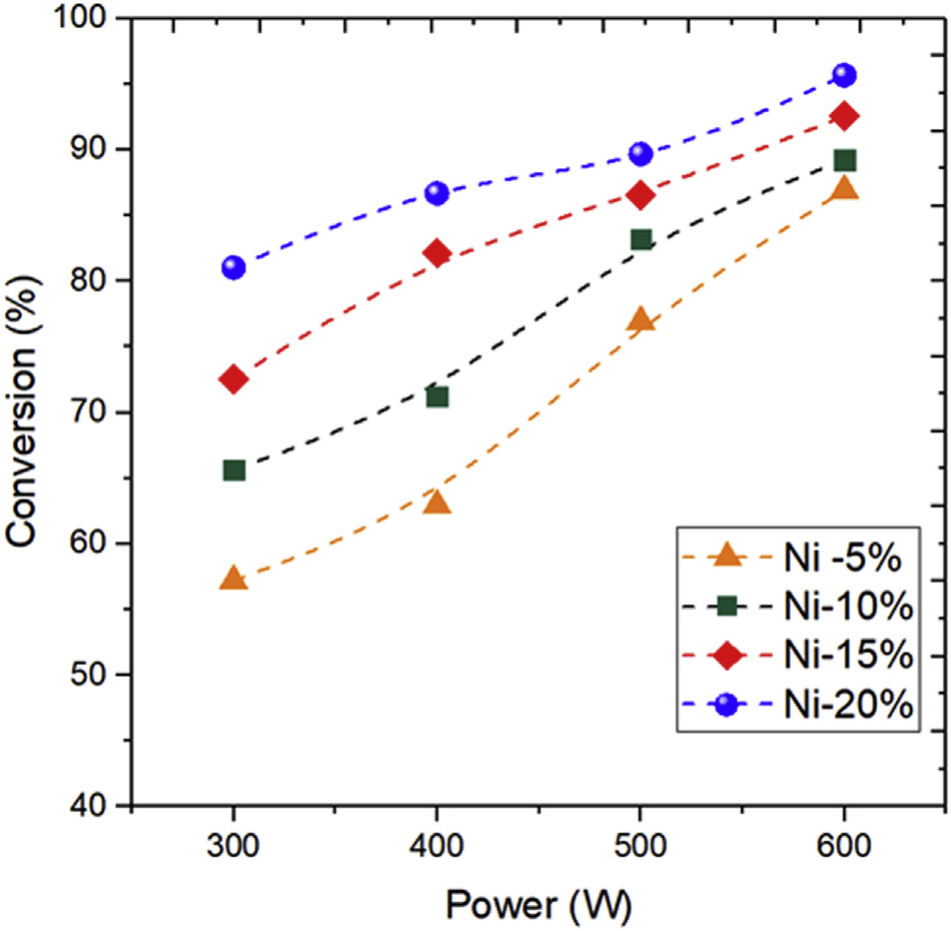
The influence of microwave power and nickel loading level on the glycerol conversion at feed flow rate of 0.5 ml/min.

Figure 7 shows the influence of MAC power on glycerol conversion at different NiO loading. It appears that the glycerol conversion increases significantly with the increase in NiO loading level and MAC power. In this reaction, glycerol also acts as an excellent “sacrificial” hydrogen source [47]. Additional nickel content on the zeolite support surface can improve the catalytic activity at the lower reaction temperature. Meanwhile, microwave power increases the microwave temperature, leading to an incredible catalytic reaction.

Higher temperatures also indicate increased reactant conversion. This fact can be due to the reaction could produce more energy so that it can speed up the reaction. Higher temperatures also indicate increased conversion. This is because at higher temperatures it produces more energies so it can speed up the reaction [48]. Nickel plays an important role in tremendously improving the catalytic activity of glycerol decomposition and H_2_ selectivity. Higher content of NiO in the zeolite generated a higher rate of hydrogen gas. Ni metal serves as an active phase (hydrogen evolution site), which provides a reaction, thereby producing hydrogen catalysis process is effective because the Ni metal can lower the activation energy. The interactions between MAC and the heterogeneous nickel catalyst particles generate electrostatic discharges that can lead to active site formation in the reaction media [49].

#### 3.2.3 Influence of NiO content on hydrogen generated at different MAC power

**Figure 8:**
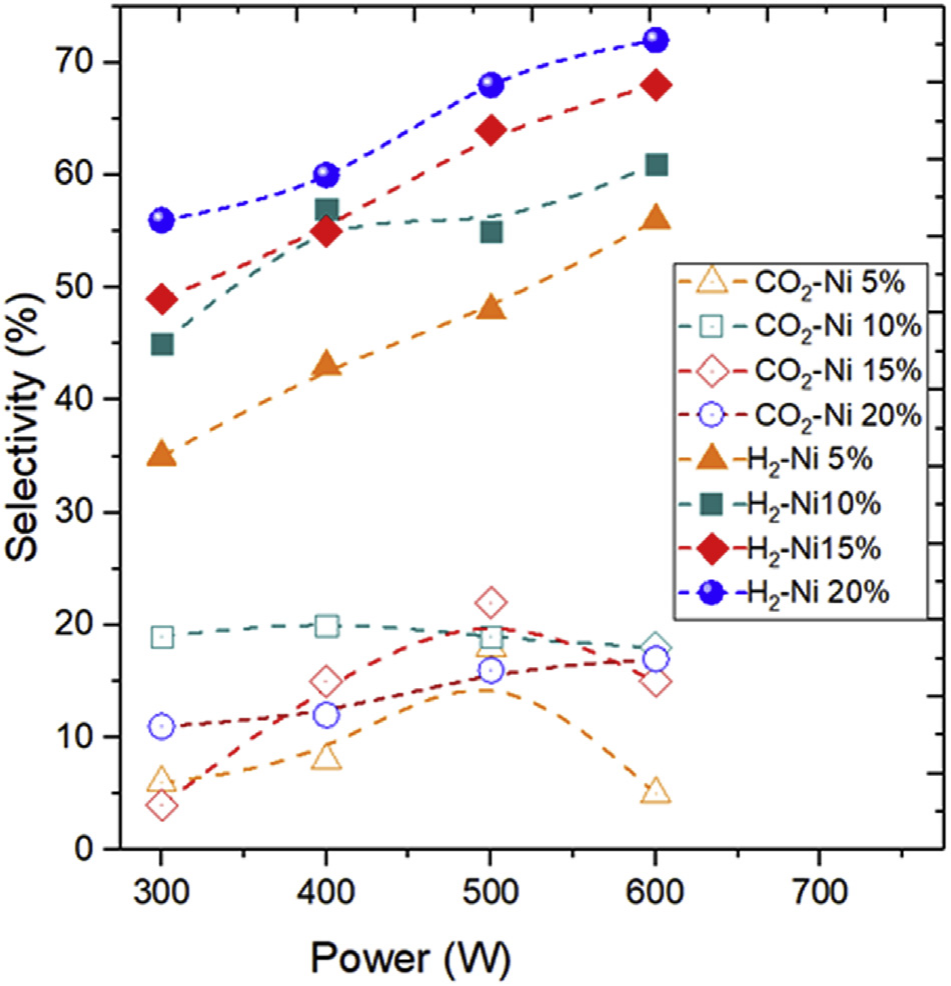
The influence of NiO content on hydrogen generated at different MAC power, and feed flow rate of 0.5 ml/min.

In the present study, we first observed the effect of the amount of nickel loading on the natural zeolite catalysts on hydrogen production. Figure 8 illustrates the results of the molar fraction of the hydrogen and CO_2_ product obtained in the tests in the range of MAC power and nickel loading on zeolite. As can be seen from Figure 8, the preparation of NiO/Zeolite was investigated at various Ni loading levels: the highest H_2_ production was obtained at a microwave power of 600 W. It can be seen that the selectivity of hydrogen at a NiO content of 5%, 10%, 15%, and 20% and a microwave power of 600 reaches 56%, 61%, 68%, and 73.5%, respectively. The NiO/Zeolite at low nickel loading show the lowest level of hydrogen production. In addition, the catalyst with a NiO content of 20% has a higher selectivity of hydrogen. This finding in accordance with reported by other group work on hydrogen production at different nickel loadings generation [50]. Additionally, the activities increased with increasing microwave power, reaching the maximum H_2_ at a microwave power of 600 W and nickel loading 20 wt%.

In contrast, the CO_2_ selectivity at a selected power did not increase with increasing microwave power. The selectivity of carbon dioxide was unstable, some appeared to be decreasing, and there was also an increase. Although CO_2_ selectivity remained constant at 20% NiO content, hydrogen production increased dramatically at higher NiO content. The selectivity of CO_2_ appeared to be stable upon the use of catalyst with 15% NiO content.

As evident on a TEM image, NiO particle shows a good dispersion on the surface support can accelerate a high catalytic activity for hydrogen production. The synergic effect of NiO catalyst loading and microwave power were the reasons that MAC irradiation as a candidate for the acceleration of the NiO/zeolite-nanoparticle-catalyzed for hydrogen generation from aqueous glycerol solution. MAC irradiation of glycerol appears to be a promising candidate for the production of H_2_ from aqueous glycerol solution.

For comparison purposes, in Figure 8 is reported the best condition was achieved at a reaction power of 600 W, feed flow rate of 0.5 ml/min, and NiO content of 20% to enhance the highest glycerol conversion and H_2_ selectivity. At this condition, the glycerol conversion achieved 96.67%, and H_2_ production reached 73.5%. This result is higher than reported by Ni/SiO_2_ catalyst was found to be active for glycerol reforming reaction (GSR) with the highest H_2_ selectivity 70% and the highest glycerol conversion to gaseous products 90% [51].

Deng et al. [37] studied pyrolysis of ethanol for syngas production of the average hydrogen concentrations over activated carbon (AC). The result shows that with the increase of microwave power, the average hydrogen level increased from 49.4 vol% at 160 W to 53.8 vol% at 320 W and 55.3 vol% at 560 W, respectively. Our result has higher hydrogen selectivity about 1.33 times than reported by Deng et al. [39].

## 4 Conclusion

The catalytic reaction of water-glycerol solution as green feedstock was successfully performed under microwave-assisted catalytic over NiO/zeolite catalyst. Nanocrystalline nickel/Zeolite particles prepared using a inception wetness method were investigated. The NiO/Zeolite had a particle size is ranging of 30–200 nm. The NiO as an active site appear a good dispersion on the entire surface of zeolite support. From TEM images, tt also indicates that the particle size of NiO around 5 nm. Rapid conversion of glycerol was achieved using microwave power, feed flow rate, and NiO loading supported with the appropriate condition. The best condition was achieved at a reaction power of 600 W, feed flow rate of 0.5 ml/min, and NiO content of 20% to enhance the highest glycerol conversion and H_2_ selectivity. At this condition, the glycerol conversion achieved 96.67% and H_2_ production achieved 73.5%. At an appropriate level, both microwave heating and NiO/Zeolite content show higher H_2_ production, while microwave heating could accelerate the reaction rate, and NiO is more effective in H_2_ generation from an aqueous glycerol solution. MW reaction should shorten the reaction time. The combining microwave heating and NiO/Zeolite catalyst produced thus show great potential for broad applications. Hopefully, this work will contribute to developing a new and green process and has strong potential to be used for fuel production.

## Declarations

### Author contribution statement

Husni Husin: Conceived and designed the experiments; Contributed reagents, materials, analysis tools or data; Wrote the paper.

Mahidin Mahidin: Contributed reagents, materials, analysis tools or data; Wrote the paper.

Komala Pontas: Conceived and designed the experiments; Analyzed and interpreted the data; Wrote the paper.

Ahmadi Ahmadi, Muhammad Ridho, Fahrizal Nasution & Fikri Hasfita: Conceived and designed the experiments; Performed the experiments.

Erdiwansyah Erdiwansyah: Analyzed and interpreted the data; Wrote the paper.

M. Hazwan Hussin: Conceived and designed the experiments; Analyzed and interpreted the data.

### Funding statement

This work was supported by the Ministry of Higher Education and Technology under Fundamental Research Grant Scheme (PDUPT) No. 215/SP2H/LT/DPRM/2020/2021.

### Data availability statement

Data included in article/supp. material/referenced in article.

### Declaration of interests statement

The authors declare no conflict of interest.

### Additional information

No additional information is available for this paper.
